# Genome-wide association study for proliferative diabetic retinopathy in Africans

**DOI:** 10.1038/s41525-019-0094-7

**Published:** 2019-08-29

**Authors:** Chang Liu, Guanjie Chen, Amy R. Bentley, Ayo Doumatey, Jie Zhou, Adebowale Adeyemo, Jinkui Yang, Charles Rotimi

**Affiliations:** 1 The Center for Research on Genomics and Global Health, National Human Genome Research Institute, National Institutes of Health, Bethesda, MD USA; 2 Department of Endocrinology, Beijing Tongren Hospital, Capital Medical University, Beijing, 10730 China; 3 Beijing Diabetes institute, Beijing, 100730 China

**Keywords:** Genome-wide association studies, Diabetes complications

## Abstract

Proliferative diabetic retinopathy (PDR) is a sight-threatening complication of diabetes that is associated with longer duration of diabetes and poor glycemic control under a genetic susceptibility background. Although GWAS of PDR have been conducted in Europeans and Asians, none has been done in continental Africans, a population at increased risk for PDR. Here, we report a GWAS of PDR among Africans. PDR cases (*n* = 64) were T2D patients with neovascularization in the retina and/or retinal detachment. Controls (*n* = 227) were T2D patients without listed eye complications despite high risk (T2D duration ≥10 years and fasting blood glucose >169 mg/dl). Replication was assessed in African Americans enrolled in the ARIC study. We identified 4 significant loci: WDR72, HLA-B, GAP43/RP11-326J18.1, and AL713866.1. At WDR72 the most strongly associated SNPs were rs12906891 (MAF = 0.071; *p* = 9.68 × 10-10; OR = 1.46, 95% CI [1.30,1.64]) and rs11070992 (MAF = 0.14; *p* = 4.23 × 10^−8^; OR = 1.28, 95%CI [1.17–1.40]). rs11070992 replicated in African Americans (*p* = 0.04). Variants in this gene have been associated with diabetic retinopathy, glycemic control, revascularization, and kidney disease.

## Introduction

Diabetic retinopathy (DR) is a well-known complication of type 1 (T1D) and type 2 diabetes (T2D). It is the most frequent cause of new cases of blindness in the working-age population^1^ and the disorder progresses from non-proliferative DR (NPDR) to sight-threatening proliferative DR (PDR). The latter is characterized by the growth of abnormal new blood vessels on the retina. Neovascularization and contraction of the accompanying fibrous tissue can distort the retina and lead to tractional retinal detachment, producing severe and often irreversible vision loss. In addition, the new blood vessels may cause pre-retinal or vitreous hemorrhage and neovascular glaucoma, which can also cause vision loss. By 2020, the prevalence of DR is expected to almost double to ~7.2 million within the USA alone.^2^ Notably, the prevalence of PDR varies considerably by ethnicity with estimates as high as 8.99% in Africans and 2.67, 1.29, and 5.10%, respectively in Chinese, South Asian and Hispanic individuals^3–7^ African ancestry individuals within the US also display higher DR prevalence and more severe DR compared to non-Hispanic Whites.^8^

Epidemiologic studies suggest that the severity of DR closely correlates with glycemic control and diabetes duration,^2,9^ and optimizing glycemic control is the main recommendation to reduce the risk or slow the progression of retinopathy.^10^ There is mounting evidence indicating a large genetic contribution to the development and severity of DR as well.^11,12^ Although the Family Investigation of Nephropathy and Diabetes (FIND)-Eye study reported a broad-sense heritability for DR of 27% overall,^13^ estimates for PDR have been reported to be as high as 52%.^14^ Furthermore, even though those with T1D may have difficulty controlling their glycemia, many individuals living with T1D for 50 years or more never develop PDR.^15,16^Together, these observations suggest that genetic factors may influence a diabetic individual’s risk of developing DR and progression to PDR.

Knowledge about the specific genetic risk factors for DR has come from genome-wide association studies (GWAS) (Table S1) including studies of Taiwanese, Mexican-American, American (European Ancestry), Chinese, and Japanese T2D patients.^2,17–22^ Despite African ancestry individuals displaying high prevalence of PDR, only one GWAS for PDR has been conducted in African Americans, and this study failed to identify any significantly associated loci.^23^

## Results

Characteristics of study participants are displayed in Table 1. Consistent with our selection criteria for controls, both duration of diabetes (AADM 15.5 years; ARIC-AA 17.3 years) and fasting blood glucose levels were high among controls (AADM 268.3 mg/dl; ARIC-AA 283.7 mg/dl). In AADM, cases also had higher triglycerides, HDL cholesterol and total cholesterol levels (*p* < 0.05). The effects of population stratification on our data were assessed through visualization of Q-Q plots (λ = 1.015) using the full set of markers (Supplementary Fig. S1) and stratified by MAF (Supplementary Fig. S2). The effects were determined to be negligible following adjustment for the first two principal components. Relatively little additional information was found beyond the first 2 PCs, which are consistent with an East/West Africa split (PC1) and a gradient across West African samples (PC2) (Supplementary Fig. S3).

**Table 1:** Characteristics of study participants

	Discovery (AADM)			Replication (ARIC-AA)		
	Cases^a^	Controls^b^	*P*-values	Cases^a^	Controls^b^	*P*-values
N (% in Female)	64 (57.81%)	227 (60.35%)	0.8248	20 (70.0%)	59 (61.0%)	0.6514
Age (Years)	59.89 (10.10)	58.08 (9.67)	0.2030	60.64 (6.04)	60.55 (5.90)	0.9522
SBP (mmHg)	147.5 (27.68)	140.4 (24.71)	0.0652	137.5 (15.71)	130.1 (22.79)	0.1135
DBP (mmHg)	83.97 (13.80)	80.20 (13.80)	0.0568	75.3 (12.92)	76.45 (11.07)	0.7226
Fasting blood glucose (mg/dL)	194.2 (74.04)	268.3 (73.88)	**<0.0001**	192.0 (89.8)	283.7 (66.92)	**0.0003**
TG (mg/dL)	191.8 (85.3)	117.3 (69.05)	**0.0067**	202.0 (132.9)	145.3 (78.85)	0.0990
HDL-C (mmol/L)	53.11 (19.32)	44.30 (16.49)	**0.0015**	52.90 (14.7)	48.14 (13.35)	0.2390
CHOL (mg/dL)	238.2 (93.81)	207.1 (57.23)	**0.0151**	229.1 (54.60)	204.3 (37.22)	0.4610
HTN Medication (%)	20 (31.25%)	100 (44.05%)	**<0.0001**	13 (65.0%)	32 (54.24%)	0.4007
Lipid-lowering medication (%)	0 (0%)	2 (0.88%)	0.451	1 (5.0%)	2 (3.39%)	0.7448
LDL-C (mmol/L)	142.8 (52.60)	139.0 (46.98)	0.6111	137.5 (47.2)	128.1 (32.9)	0.4610
BMI (kg/m^2^)	26.11 (4.15)	25.26 (4.22)	0.1529	34.48 (7.43)	32.05 (6.05)	0.2000
Duration of T2D (Years)	8.28 (5.87)	15.45 (5.91)	**<0.0001**	19.93 (8.05)	17.34 (7.83)	0.2700

For continuous variables, values are given as the mean (standard deviation)

*P*-value < 0.05 for comparisons between cases and controls indicated with bold font

^a^Cases were defined as type 2 diabetes patients with PDR

^b^Controls were defined as patients who have a fasting blood glucose level ≥169 mg/dl, T2D duration duration ≥10 yrs and NDR

**Fig. 1:**
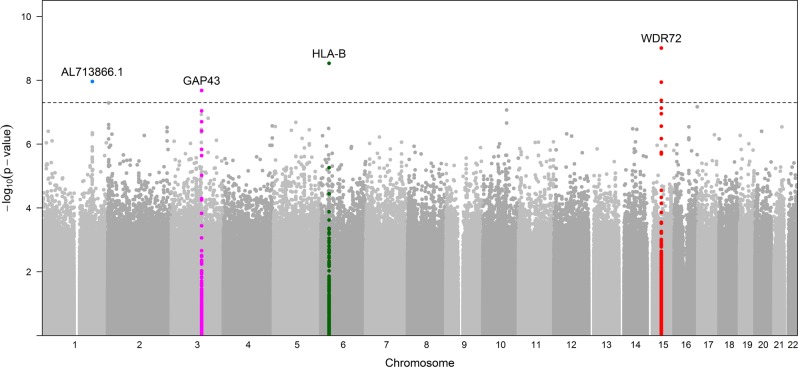
GWAS Manhattan plot for PDR in the AADM study. The dotted line represents −log10 (5 × 10^−8^)

**Fig. 2:**
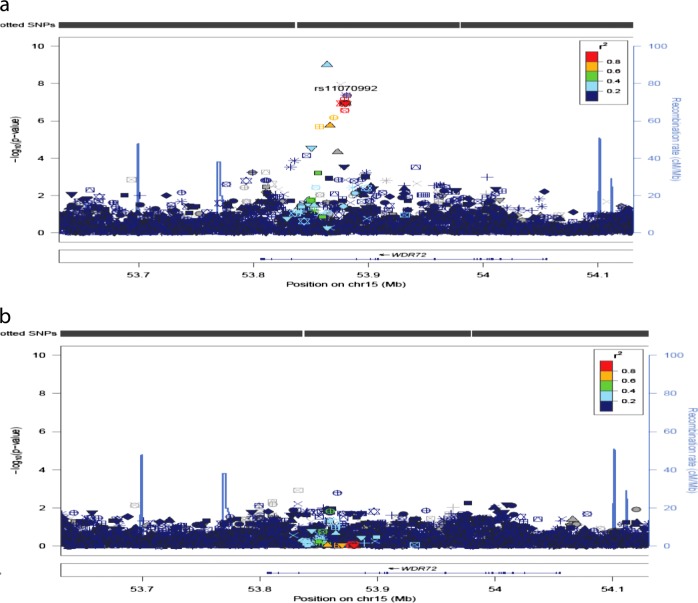
Regional plots for association and conditional analyses on rs11070992 in *WDR72*, using AFR from 1000 Genomes data^60^ as an LD reference. On the left y-axis is the -log10(*p*-value) for each SNP genotyped. On the right y-axis are the recombination rates estimated from the 1000 genome project. The annotated genes in the region are indicated along the bottom of the figure. Panels show the regional associations before (**a**) and after (**b**) adjusting for rs11070992

Association results for PDR showed 7 variants in 4 loci that reached genome-wide significance (*p* < 5 × 10^−8^) in the discovery sample (Table 2; Fig. 1). Four of these SNPs were in intron 1 in *WDR72*, 1 SNP in HLA-B, 1 SNP in *AL713866.1* and 1 in *GAP43*. The most statistically significant association was for the T allele for rs12906891 (*p* = 9.68 × 10^−10^, OR = 1.46 [1.30, 1.64]) located in intron 1 of *WDR72* on chromosome 15. Two other *WDR72* variants (rs11070992 and rs67619978), in linkage disequilibrium (LD) with rs12906891, which have the *r*^2^ in AADM and ARICAA were 0.384/0.381 and 0.518/0.504, respectively, were also genome-wide significant. In AADM the LD of rs11070992 and rs67619978 is high, *r*^2^ = 0.98 and the *r*^2^ of rs11070992 and rs3081219 is 0.87. Population frequency data for rs3081219 is not available. Analysis conditioned on rs11070992 showed only one peak in this region (Fig. 2).

**Table 2:** Genome-wide significant SNPs among Africans and replication among ARIC African Americans

				AADM						ARIC-AA					Meta-analysis		
SNPs	Genes	Chr:Pos (bp)	Effect/Ref. Alleles	EAF/N	OR	95% CI	Genotype Counts	*P*-values	Info score	EAF/N	OR	95% CI	*p*-values	Info score	OR	95% CI	*P*-values
rs12906891	*WDR72*	15:53864144	T/C	0.07/291	1.46	1.30,1.64	Ca: 38/24/2 Co: 214/13/0	9.7E−10	0.94	0.14/79	1.10	1.41,1.93	0.41	0.96	1.38	1.24,1.53	1.6E−09
rs3081219	*WDR72*	15:53876165	C/CAG	0.13/291	1.31	1.19,1.44	Ca: 30/31/3 Co: 193/32/2	1.1E−08	0.98	0.18/79	1.22	0.87,1.40	0.08	0.99	1.29	1.19,1.41	1.0E−09
rs11070992	*WDR72*	15:53880517	G/A	0.14/291	1.28	1.19,1.39	Ca: 30/30/4 Co: 188/37/2	4.2E−08	0.96	0.19/79	1.25	0.98,1.53	0.04	0.99	1.28	1.18,1.38	2.0E−09
rs67619978	*WDR72*	15:53881471	A/ACACGAATT TCAGTTTGTA	0.14/291	1.28	1.19,1.39	Ca: 30/30/4 Co: 188/37/2	4.2E−08	0.95	0.18/79	1.24	1.02,1.55	0.04	0.98	1.27	1.18,1.38	2.1E−09
rs1065386	*HLA-B*	6:31324547	C/G	0.43/291	1.21	1.14,1.28	Ca: 3/36/25 Co: 89/111/27	3.0E−09	0.86	0.37/79	0.91	0.77,1.08	0.30	0.79	1.17	1.11,1.24	5.2E−08
rs10560003	*GAP43/RP11-326J18.1*	3:115378465	T/TAGAG	0.02/291	1.86	1.50,2.31	Ca: 55/8/1 Co: 227/0/0	2.1E−08	0.85	0.04/79	0.98	0.71,1.33	0.88	0.90	1.52	1.28,1.81	2.5E−06
rs72740408	*AL713866.1*	1:191105831	A/G	0.03/291	1.65	1.41,1.93	Ca: 47/16/1 Co: 225/2/0	1.1E−08	0.74	0.06/79	1.13	0.82,1.56	0.45	0.94	1.52	1.31,1.76	2.3E−08

Replication in ARIC-AA was attempted for each of the variants that were genome-wide significant in the discovery sample of Africans. Of these, 2 were *p* < 0.05 and in the same direction of association among African Americans, both of which were *WDR72* variants (in linkage disequilibrium with each other [*r*^2^ = 0.9]).

To further evaluate the variants that were genome-wide significant in the AADM analysis, we conducted several secondary analyses. To determine the extent to which our control selection enabled the detection of these loci, we also ran analyses using any type 2 diabetic participant without PDR as a control. For 6 of the 7 variants tested, the statistical significance was reduced, despite the larger sample size for the controls using this strategy (Table S2). The exception was rs72740408 (*AL713866.1*), which was more statistically significant using T2D controls, though the effect was reduced (extreme: OR 1.86, *p* = 1.1E−8; T2D: OR 1.15, *p* = 1.6E−11). We also tested the lead SNPs from the PDR association for an association with fasting blood glucose among AADM participants (stratified by case status, given the effect of medication on glucose values); one of the variants tested reached nominal significance (rs12906891 in *WDR72*: beta −0.07, *p* = 0.03; Table S3). Adding glucose as a covariate to the analysis of PDR slightly reduced the statistical significance of the results but does not appear to fully mediate the association with PDR (*p*-values of lead SNPs in adjusted analysis: 6.2E−6 to 3.3E−8; Table S4).

The HLA-B variant that was genome-wide significant in the analysis of Africans (rs1065386), produces a missense change at position 87 of the amino acid sequence that is found on several classical HLA-B alleles, including HLA-B*07:02, HLA-B*07:05, HLA-B*14:01, and HLA-B*35:01, among others. We tested the classical HLA-B alleles that include the rs1065386 variant allele for an association with PDR in our data (Table S5). HLA-B*07:02 (*p* = 0.016) and HLA-B*45:01 (*p* = 0.017) were nominally significantly associated with PDR in the AADM data.

We also investigated previously reported top associations for PDR in our data, though few of these associations reached genome-wide significance in the original publications (Supplementary Table S6). In both AADM and ARIC-AA, we replicated rs1902491 (OR = 0.91/0.85, *p* = 0.0013/0.02) near *NPY2R/LOC729902*, which was reported in three different cohort studies^2^. In AADM, we replicated rs918519 (OR = 0.913, *p* = 0.014) near *LOC285626*, which was recently reported in White Australian individuals.^22^ While our replication supports these previous findings, neither of these associations were GWAS significant in the original publication, and they would not reach this threshold with meta-analysis with our data.

## Discussion

Despite evidence for a genetic contribution to proliferative diabetic retinopathy, GWAS have not been very successful in identifying loci for PDR. Many of the results in previously published GWAS of PDR did not reach genome-wide significance (Supplementary Table S1), and the top loci from these studies have not been replicated in independent populations.^2,17,20,24,25^ Results from candidate gene association studies have also been conflicting.^26^ These studies vary considerably in case-control and outcome definition. In our study, we use an “extreme phenotype” approach. As diabetic retinopathy is closely related to glycemic level and diabetes duration, ^2,9^ we chose controls who had at least 10 years T2D duration and high glycemia but with no evidence of DR. By selecting controls that had sufficient environmental risk to make them susceptible to PDR, we decreased the chance that any of the controls had genetic risk factors for PDR: under these conditions, individuals with predisposing genetic factors for PDR would be expected to have developed it. Based on this careful definition of controls, this first GWAS of PDR in continental Africans with T2D identified 4 genome-wide significant loci with replication of one of these (*WDR72*) in African Americans.^27^

*WDR72* (WD repeat-containing protein 72,15q21.3) is a protein-coding gene that has been associated with HbA1c level and poor glucose control.^28^ Notably, *WDR72* mRNA is expressed in the retina.^28^ Diabetic kidney disease (DKD) and DR are both microvascular complications of T2D and share pathophysiological mechanisms and common risk factors.^25^ Some DKD studies require the presence of DR in controls, highlighting the correlation between these outcomes.^25^ *WDR72* is associated with eGFR and is highly expressed in the kidney epithelium, suggesting that genetic variation at these loci may have relevance for both microvascular complications.^28,29^ An important paralog of *WDR72*, *WDR7*, is associated with retinitis pigmentosa 4, a highly expressed protein in the retina (https://www.genecards.org/cgi-bin/carddisp.pl?gene=WDR7). Additionally, WDR72 is critical to ameloblast vesicle turnover during enamel maturation.^30^ Enamel matrix derivative induces proliferation, viability, and angiogenesis of human microvascular cells.^31^ These observations suggest WDR72 may be involved in proliferative angiogenesis of the microvasculature in the eyes.

HLA-B (Major Histocompatibility complex class 1, B) is a protein-coding gene which is expressed in the retina and the pancreas. HLA-B was also associated with DR in African and Caucasian in T1DM.^32,33^ In terms of classical HLA alleles, the HLA-B alleles showing significant association with PDR in the present study are HLA-B*07:02 (*p* = 0.0155) and HLA-B*45:01 (*p* = 0.0171). These are not the same classical HLA-B alleles reportedly associated with DR in T1DM: HLA-B49^32^ and HLA-B62.^33^

*GAP43* (growth-associated protein 43, gene ID:172020, 3q13.31) is a protein-coding gene which is expressed in the retina and pancreas. GAP43 is also an axonal regenerative marker which is related to diabetic peripheral neuropathy, while axons and blood vessels share molecular signals for purpose of navigation, regeneration, and terminal arborizations.^34^ The function of rs72740408 located near RNA gene *AL713866.1* is still unknown.

We also replicated previous reported PDR associations with rs1902491 (near *NYP2R*) and rs918519 (near *LOC285626*). Neuropeptide Y Receptor subtype 2(NPY2R) is a G protein-coupled receptor for NPY, a neurotransmitter released by endothelial cells implicated in ischemic angiogenesis.^35^ There is substantial genetic, biologic, and functional data supporting a role for neuropeptide Y signaling in diabetic retinopathy.^36–39^ rs918519 (35 kb upstream of *LOC285626*) has been previously associated with PDR.^22^ This gene encodes an uncharacterized, long non-coding RNA. The nearest protein-coding gene is *IL12B* (Interleukin 12B), a further 33 kb upstream. *IL12B* is expressed in the retina (The Ocular Tissue Database^40^) and has been implicated in both T1D^41–43^ and T2D.^44^

To date, only one other study has used a similar control selection strategy (only including diabetics with a long duration of disease). In that study, Shtir et al.^45^ performed whole-exome sequencing on 64 cases and 43 controls of Saudi descent. They identified three genes, *NME3*, *LOC728699*, and *FASTK*, whose increased rare variant burden appeared to protect against DR. We did not find association in these genes in our study, which may reflect our focus on common variants.

A potential limitation of this study is the small sample size which is partly due to the strict definition of controls to include only those at high risk that did not develop PDR; however, we believed this approach which resulted in “cleaner phenotypic classification” enhanced our ability to identify GWAS significant loci for PDR. When we evaluated the identified associations in an analysis that used all diabetics as controls instead of our extreme control design, we see a diluted association for all but one variant, consistent with the less carefully defined controls adding uncertainty to the analysis, as some of the controls may have become cases with sufficient length of T2D diagnosis or poor glucose control. It is anticipated that a greater number of subjects, using this strategy to define control subjects, will yield even more loci. Our discovery sample consisted of continental Africans, while our replication set was African Americans. While it would have been ideal to conduct replication in Africans, as well, we are unaware of other datasets of Africans in which such an analysis could be conducted. Thus, it is uncertain whether our inability to replicate some of our findings was a result of false positives or other factors including small replication sample size, or differences in genomic (e.g., admixture) or environmental context between Africans and African Americans.

In all, this GWAS of PDR among Africans identified 4 genome-wide significant loci, one of which replicated among African Americans. Identified loci highlight pathways of known significance to diabetic retinopathy, including glycemic control, and revascularization.

## Methods

### Ethics statement

The AADM study was approved by the Institutional Review Board (IRB) at the National Institutes of Health, and the ARIC study was approved by the IRB of all participating institutions, including the IRBs of the University of Minnesota, Johns Hopkins University, University of North Carolina, University of Mississippi Medical Center, and Wake Forest University. Both studies were conducted in accordance with the Declaration of Helsinki. Written informed consent was obtained from all human participants.

### Study design

Study individuals were drawn from the AADM study, a large, ongoing genetic epidemiology study of T2D and related traits in Africans.^46^ Demographic information was collected using standardized questionnaires across the AADM study centers. Anthropometric, medical history, and clinical examination parameters were obtained by trained study staff during a clinic visit. Blood samples were drawn after an overnight fast of at least 8 h. The definition of T2D was based on the American Diabetes Association (ADA) criteria.^47^ Biochemical parameters were measured with a Beckman-UniCel Dxc 800 biochemistry analyzer (Beckman Coulter, Carlsbad, CA), including fasting plasma glucose, total cholesterol, triglycerides, LDL and HDL cholesterol.

### Evaluation of PDR

All AADM study participants underwent a thorough ophthalmological examination. The eye examination was part of a comprehensive physical examination of each participant in the study. Each participant had the following ocular examinations: visual acuity; ocular alignment and motility; pupil reactivity and function; visual fields; intraocular pressure; slit lamp examination of the cornea, iris, lens, and vitreous; and dilated fundus examination. Previous experiences of the ophthalmologists in the 5 centers demonstrate a better than 80% concurrence between their clinical and photographic assessment of the presence and absence of lesions associated with diabetic retinopathy. To assure reproducibility of the assessment and classification of ocular complications between the 5 centers, the absence and/or presence of hemorrhages, microaneurysms, cotton wool spots, neovascularization, cataracts, retinal detachment, maculopathy, and glaucoma in each subject’s eyes was recorded, along with other ocular abnormalities.^48^ A diagnosis of PDR was made only with neovascularization in any field or retinal detachment with a diagnosis of diabetes retinopathy.

### Selection of PDR cases and controls

Patients with PDR (neovascularization and or retinal detachment) were selected as cases, irrespective of glucose level and duration of diabetes.

While most genetic studies of PDR to date have used a general sample of T2D patients as the control group, this approach runs the risk of misclassifying T2D patients without a long history of disease or with good glycemic control as controls as they might develop disease if challenged with poor glucose control or with a longer time since diagnosis. To minimize such misclassification bias, we selected as controls only those patients with fasting blood glucose of at least 169 mg/dl (which is equivalent to HbA1C > 7.5% [58 mmol/mol]) and with a duration of diabetes of at least 10 years but without diabetic retinopathy (NDR). We reasoned that using such “super-controls” will improve the power of the study through more precise phenotype definition, albeit at the cost of a reduction in sample size.

### Genotyping and imputation

A total of 5231 African individuals from the AADM study were genotyped on high density GWAS arrays (Affymetrix® Axiom® Genome-Wide PanAFR and Illumina Multi-Ethnic Genotyping arrays. Comparable proportions of cases were genotyped on each array (Affymetrix: 22.9%, Illumina: 21.1%).

After technical quality control and appropriate sample- and SNP-level filtering (individual call rate ≤95%, SNP call rates ≤95%, Hardy–Weinberg *p* < 10-6, and minor allele frequency (MAF) < 0.01), imputation was performed using the African Genome Resources Panel available from the Sanger Imputation Service (https://imputation.sanger.ac.uk/).^49^ The African Genome Resources Imputation Reference Panel comprised 4956 individuals, including all 2504 from the 1000 Genomes Project Phase 3, ~2000 individuals from Uganda (Baganda, Banyarwanda, Barundi, and others), and ~100 individuals from each of a set of populations from Ethiopia (Gumuz, Wolayta, Amhara, Oromo, and Somali), Egypt, Namibia (Nama/Khoe-San) and South Africa (Zulu), yielding 9912 haplotypes for 93,421,145 SNPs. Pre-phasing was performed with EAGLE version 2.0.5^50^ and imputation was performed using software Positional Burrows-Wheeler Transform (PBWT).^51^ The resulting imputation dataset was filtered for MAF ≥ 0.01 and info ≥ 0.3. A total of 18,433,741 SNPs was tested. Coordinates are given based on the hg19 genome build.

### Association analysis

Association analysis was performed using the EPACTS 3.2.6 (Efficient and Parallelizable Association Container Toolbox) pipeline (http://genome.sph.umich.edu/wiki/EPACTS), using imputed dosages and adjusting for genetic relatedness, sex, age, and the first two principal components. Within EPACTS, we performed single variant EMMAX association analysis. Genome-wide significance was declared to be *p* < 5 × 10^−8^. Q-Q and Manhattan plots were generated using R 3.4.2 (http://www.r-project.org).

Secondary analyses were conducted to further characterize the top SNPs of our main analysis (all *p* < 5 × 10^−8^). To evaluate our control selection strategy, we also ran analyses as above, but included as controls all AADM participants with T2D, but no PDR, regardless of duration of T2D or presence of hyperglycemia (*n* = 1724). To determine whether these variants were associated with PDR through an association with glucose, we also ran our main analysis model with the addition of glucose as a covariate. Additionally, we tested these variants for an association with glucose, separately in AADM participants with (*n* = 2048) and without T2D (*n* = 2133; to avoid effects of treatment on glucose concentration). We further evaluated a finding in the HLA-B region by conduct HLA imputation using the HIBAG algorithm^52^ and show that the missense change is on several classic HLA-B alleles.

### Replication study and meta-analysis

The replication study was conducted in the Atherosclerosis Risk in Communities (ARIC) study accessed through dbGaP (phs000280.v2. p1). ARIC was designed as a multi-ethnic study of atherosclerosis and recruited individuals in Forsyth County, NC; Jackson, MS; the suburbs of Minneapolis, MN; and Washington County, MD. It is a population-based study of 15,792 individuals aged 45–64 years at their first examined in 1987–1989.^53^ A second examination was conducted in 1990–1992 and a third in 1993–1995 when retinal photographs were taken on all participants.^54^ Our analysis only includes AAs of the third phase of ARIC. One randomly selected eye was photographed using a nonmydriatic camera and evaluated by masked graders according to standardized protocols, as has previously been described,^54,55^ allowing for consistent PDR definitions with AADM. Individuals from ARIC were genotyped using the Affymetrix® Genome-Wide SNP Array 6.0.^53^ For the ARIC data, imputation was performed using impute2 software and 1000 genome phase 3 panel from the Sanger imputation service (https://imputation.sanger.ac.uk/). The resulting imputation dataset was filtered for MAF ≥ 0.01 and info ≥ 0.3.

Cases and controls were defined as in the discovery study. Replication was assessed in 19 cases and 50 controls obtained from the ARIC African Americans study (ARIC-AA; *n* = 3137).^56^ Imputation and association analysis were performed as described above. We adjusted for genetic relatedness, sex, age, and the first two principal components. METAL^57^ was used to perform inverse variance-weighted fixed-effect meta-analysis of the discovery and replication samples.

### Regional plot and conditional analysis

Association results for PDR at a 250-kb region surrounding rs11070992(*WDR72*) were plotted using Locus Zoom (http://www.locuszoom.org). Conditional analysis was conducted using PLINK 1.9.^58^

### Statistical analysis

Demographic and clinical characteristics were compared using chi-squared tests for dichotomous traits and t-tests for continuous traits. R-studio was used for preparation of the phenotype data and annotation.

To minimize the potential effect of population structure, we adjusted all analyses by principal components obtained from the R package SNPRelate,^59^ which uses genotyped SNPs to generate a genetic covariance matrix followed by eigen decomposition.

### Reporting summary

Further information on research design is available in the Nature Research Reporting Summary linked to this article.

## Supplementary information

Supplementary Materials

Reporting Summary Checklist

## Data Availability

The datasets generated during and/or analyzed during the current study are not publicly available due to the inconsistency of study consent documents with repository deposition but are available from the corresponding author upon reasonable request.

## Supplementary information

**Supplementary Information** accompanies the paper on the *npj Genomic Medicine* website (10.1038/s41525-019-0094-7).
