# Supplementary material for: Genome-wide association study for proliferative diabetic retinopathy in Africans
Source: NPJ Genom Med. 2019 Aug 29;4:20. doi: 10.1038/s41525-019-0094-7 (PMC6715701; doi:10.1038/s41525-019-0094-7)
Supplement: Supplementary file 1 — Supplementary Materials [file 41525_2019_94_MOESM1_ESM.pdf]

Supplementary Material:
Genome-wide association study for proliferative diabetic retinopathy in Africans

Chang Liu[1,2,3,] Guanjie Chen[1], Amy R. Bentley[1], Ayo Doumatey[1], Jie Zhou[1], Adebowale Adeyemo[1], Jinkui Yang[2,3] & Charles Rotimi[1]

[1]The Center for Research on Genomics and Global Health, National Human Genome Research Institute, National Institutes of Health, Bethesda, Maryland USA

[2]Department of Endocrinology, Beijing Tongren Hospital, Capital Medical University, Beijing 10730, China

[3]Beijing Diabetes institute, Beijing 100730, China

Supplemental Materials **Page**

Table S1. Published genome- wide association studies for diabetic retinopathy 2

Table S2. Comparison of Results of Analyses with Extreme Controls (with Hyperglycemia and Long T2D Duration) and T2D Controls 3

Table S3. Association of Lead SNPs from AADM PDR GWAS with Fasting Glucose 4

Table S4. Association of Lead SNPs with PDR with Adjustment for Fasting Glucose 5

Table S5. Association of HLA-B Classical Alleles that include rs1065386 missense change with PDR 6

Table S6. Replication of published findings for PDR in Africans and African Americans 7

Figure S1. Quantile-quantile of p values for PDR 9

Figure S2. Quantile-quantile of p values for PDR stratified by allele frequency 10

Figure S3. Population Structure in AADM and ARIC-AA 11

Table S1: Published Genome-wide Association Studies of Diabetic Retinopathy

**Published studies/ Genotyping platform**	**Ancestry Populations**	**Diabetes Type**	**Case definition**	**Control definition**	**Significant SNPs (gene)**
Fu *et al., J Ophthal* 2010/ Affymetrix 100k	Hispanic	2	Moderate to severe NPDR and PDR	No DR to early NPDR	none
Grassi *et al., Hum Mol Genet* 2011/ Affymetrix5.0 and Illumina HapMap550	European	1	PDR and DME	NPDR and DR	none
Huang *et al. Ophthalmology* 2011/ Illumina HapMap550	Asian	2	NPDR and PDR	NDR and Nondiabetic patients	rs13163610 (*KIAA0825*), rs17376456 (*KIAA0825*), rs12219125 (*ARHGAP22*), rs4838605 (*ARHGAP22*), rs2038823 (*HS6ST3*)
Sheu *et al., Hum Mol Genet* 2013/ Illumina OmniExpress	Asian	2	PDR	Diabetes ≥ 8 years with no DR	none
Awata *et al., PLOS One* 2014/ Affymetrix GeneChip 6.0 microarrays	Asian	2	DR	Diabetics with no DR	none
Burdon *et al., Diabetologia* 2015/ Human OmniExpress Beadchips	White Australian	2	severe NPDR, PDR, and macular edema	No or minimal DR	none, meta-analysis found rs9896052 (*GRB2*)
Tandon *et al., Invest Ophth Vis Sci* 2015/ Affymetrix 6.0 platform	African	2	PDR	No DR or NPDR	none
Graham *et al., BMC Medical Genetics* 2018/ OmniExpress SNParray	White Australian	2	PDR or DME	No DR or DME	none
Pollack *et al., Diabetes* 2019/ affy6.0, illum370, illum OmniExpress	European and African	2	(1) DR, (2) PDR, (3) NPDR/PDR, (4) PDR	(1) No DR, (2) no PDR, (3) no DR, (4) no DR	none
Meng *et al., Acta Ophthalmologica* 2018/ Affymetrix SNP6.0 chips	European	2	Severe NPDR, PDR, and treated by laser photocoagulation	No DR or mild NPDR	rs3913535 (*NOX4*)

Abbreviations: Diabetic Macular Edema (DME), Diabetic Retinopathy (DR), Non-proliferative Diabetic Retinopathy (NPDR), and Proliferative Diabetic Retinopathy (PDR).

Table S2. Comparison of Results of Analyses with Extreme Controls (with Hyperglycemia and Long T2D Duration) and T2D Controls

	Extreme Controls			T2D Controls		
	OR	95% CI	P-Value	OR	95% CI	P-Value
	Top Findings from Extreme Control Design					
rs12906891	1.46	1.30,1.64	9.68E-10	1.06	1.03,1.08	7.42E-07
rs3081219	1.31	1.19,1.44	1.14E-08	1.04	1.022,1.06	8.34E-06
rs11070992	1.28	1.19,1.39	4.23E-08	1.04	1.02,1.06	1.85E-05
rs67619978	1.28	1.19,1.39	4.23E-08	1.04	1.02,1.05	2.68E-05
rs1065386	1.28	1.19,1.39	3.0E-09	1.03	1.02,1.05	3.43E-07
rs10560003	1.21	1.14,1.28	2.1E-08	1.08	1.04,1.13	2.59E-04
rs72740408	1.86	1.50,2.31	1.1E-08	1.15	1.10,1.20	1.60E-11

Table S3. Association of Lead SNPs from AADM PDR GWAS with Fasting Glucose in T2D Cases and Controls

SNP	Gene	Chr:Pos	N	EAF	Beta	SE	P-value
T2D Controls							
rs12906891	*WDR72*	15:53864144	2133	0.07	0.06	0.03	0.07
rs3081219	*WDR72*	15:53876165	2133	0.12	0.03	0.02	0.18
rs11070992	*WDR72*	15:53880517	2133	0.14	0.03	0.02	0.28
rs67619978	*WDR72*	15:53881471	2133	0.14	0.02	0.02	0.36
rs1065386	*HLA-B*	6:31324547	2133	0.40	-0.01	0.02	0.45
rs10560003	*GAP43/RP11-326J18.1*	3:115378465	2133	0.02	-0.08	0.06	0.19
rs72740408	*AL713866.1*	1:191105831	2133	0.02	-0.05	0.06	0.42
T2D Cases							
rs12906891	*WDR72*	15:53864144	2048	0.08	-0.07	0.03	0.03
rs3081219	*WDR72*	15:53876165	2048	0.14	-0.03	0.02	0.14
rs11070992	*WDR72*	15:53880517	2048	0.15	-0.03	0.02	0.15
rs67619978	*WDR72*	15:53881471	2048	0.15	-0.03	0.02	0.16
rs1065386	*HLA-B*	6:31324547	2048	0.40	0.005	0.02	0.75
rs10560003	*GAP43/RP11-326J18.1*	3:115378465	2048	0.02	-0.07	0.06	0.21
rs72740408	*AL713866.1*	1:191105831	2048	0.02	0.01	0.06	0.85

Table S4. Association of Lead SNPs with PDR with Adjustment for Fasting Glucose

	Main Analysis			Adjusted for Glucose		
	OR	95% CI	P-Value	OR	95% CI	P-Value
	Top Findings from Extreme Control Design					
rs12906891	1.46	1.30,1.64	9.68E-10	1.38	1.23,1.55	8.45E-08
rs3081219	1.31	1.19,1.44	1.14E-08	1.24	1.14,1.36	2.33E-06
rs11070992	1.28	1.19,1.39	4.23E-08	1.22	1.12,1.33	6.21E-06
rs67619978	1.28	1.19,1.39	4.23E-08	1.22	1.12,1.33	6.21E-06
rs1065386	1.28	1.19,1.39	3.0E-09	1.18	1.12,1.25	4.68E-08
rs10560003	1.21	1.14,1.28	2.1E-08	1.74	1.42,2.12	1.69E-07
rs72740408	1.86	1.50,2.31	1.1E-08	1.58	1.35,1.85	3.29E-08

Table S5. Association of HLA-B Classical Alleles that include rs1065386 missense change with PDR

Allele	Freq. case	Freq. control	Freq[1]	P_Logit[2]	OR	L95	U95
B*07:02	0.0234	0.0698	0.0591	**0.0155***	0.2273	0.0685	0.7543
B*15:03	0.0703	0.0581	0.0609	0.3697	1.4623	0.6375	3.3546
B*35:01	0.0469	0.0814	0.0735	0.4580	0.6880	0.2562	1.8473
B*42:01	0.0391	0.0581	0.0538	0.8638	0.9102	0.3107	2.6662
B*45:01	0.0781	0.0442	0.0520	**0.0171***	3.0038	1.2158	7.4212
B*49:01	0.0859	0.0395	0.0502	0.1926	1.7831	0.7470	4.2561
B*53:01	0.0781	0.1442	0.1290	0.5196	0.7765	0.3596	1.6767
B*58:01	0.0703	0.0791	0.0771	0.5859	0.7927	0.3436	1.8286

Association done under an additive genetic model and adjusting for age, sex and the first two PCs of the genotypes. [1] Nominally significant p-values ($p<0.05$) are shown in bold. [2] Only classical alleles with frequency of 5% or higher shown.

Table S6: Replication of published findings for PDR in Africans and African Americans

Published studies	Population/Type of Diabetes/Study	Reported SNPs	Effect Allele	Gene/near gene	P-values[1]	OR[2]	95%CI
Grassi et all (2012)	European/T1DM/EDIC	rs1902491	A	near *NPY2R/LOC729902*	**0.034**	0.75	NR
	European/T1DM/GoKinD				**0.0003**	0.62	NR
	European/T1DM/WESDR				0.13	0.81	NR
Our study	African/T2DM/AADM				**0.0013**	0.91	[0.85,0.96]
Our study	African American/T2DM/ARICAA				**0.022**	0.85	[0.74,0.97]
Grassi et all (2012)	European/T1DM/EDIC	rs4865047	T	*CEP135*	**3.36E-06**	0.44	NR
	European/T1DM/GoKinD				0.26	0.87	NR
	European/T1DM/WESDR				0.11	0.65	NR
Our study	African/T2DM/AADM				0.86	1.01	[0.92,1.11]
Our study	African American/T2DM/ARICAA				0.71	1.04	[0.85,1.27]
Grassi et all (2012)	European/T1DM/EDIC	rs476141	T	near *AKT3/ZNF238*	**1.59E-05**	1.72	NR
	European/T1DM/GoKinD				**0.0015**	1.27	NR
	European/T1DM/WESDR				0.7	0.95	NR
Our study	African/T2DM/AADM				0.44	1.04	[0.95,1,13]
Our study	African American/T2DM/ARICAA				**0.024**	0.87	[0.73,1.04]
Graham et al.(2018)	European/T1DM/WESDR	rs918519	T	near *LOC285626*	**3.87E-06**	0.35	[0.22,0.54]
Our study	African/T2DM/AADM		A		**0.014**	0.91	[1.02,1.18]
Our study	African American/T2DM/ARICAA		A		0.59	1.04	[0.89,1.22]
Graham et al.(2018)	European/T1DM/WESDR	rs1158314	G	NRXN3	**4.01E-06**	2.16	[1.56,3.00]
Our study	African/T2DM/AADM		G		0.79	1.01	[0.94,1.09]
Our study	African American/T2DM/ARICAA		G		0.058	1.16	[1.00,1.34]
Graham et al.(2018)	European/T1DM/WESDR	rs8004963	C	NRXN3	**4.01E-06**	2.16	[1.56,3.00]
Our study	African/T2DM/AADM		C		0.74	1.01	[0.95,1.08]
Our study	African American/T2DM/ARICAA		C		0.055	1.15	[1.00,1.33]
Graham et al.(2018)	European/T1DM/WESDR	rs918520	C	near *LOC285626*	**6.66E-06**	0.34	[0.21,0.54]
Our study	African/T2DM/AADM		G		0.058	1.08	[0.10,1.16]
Our study	African American/T2DM/ARICAA		G		0.65	0.96	[0.81,1.14]
Grassi et all (2011)	European/T1DM	rs238250	A		**7.70E-06**	1.66	NR
Our study	African/T2DM/AADM		T		**0.043**	0.93	[0.86,0.10]
Our study	African American/T2DM/ARICAA		T		0.38	0.92	[0.78,1.10]
Burdon et all.(2015)	White Austrilian/T2DM	rs6516749	T	*ADAMTS5*	**2.76E-06**	0.50	[0.38,0.67]
Our study	African/T2DM/AADM		T		**0.0018**	1.27	[1.10,1.48]
Our study	African American/T2DM/ARICAA		T		0.15	1.20	[0.94,1.53]
Burdon et all.(2015)	White Austrilian/T2DM	rs3805931	A	*PTK7*	**2.66E-07**	0.50	[0.39,0.65]
Our study	African/T2DM/AADM		T		0.46	1.02	[0.96,1.09]
Our study	African American/T2DM/ARICAA		T		0.15	0.90	[0.78,1.04]
Burdon et all.(2015)	White Austrilian/T2DM	rs6128541	C	*EDN3*	**2.43E-06**	1.95	[1.48,2.58]
Our study	African/T2DM/AADM		C		0.86	0.99	[0.93,1.06]
Our study	African American/T2DM/ARICAA		C		0.079	1.14	[0.99,1.30]
Burdon et all.(2015)	White Austrilian/T2DM	rs9896052	A	near *GRB2*	**6.55E-05**	1.67	[1.30,2.15]

Our study	African/T2DM/AADM		C		0.56	0.95	[0.81,1.12]
Our study	African American/T2DM/ARICAA		C		0.62	0.95	[0.77,1.17]
Huang et all.(2011)	Asian/T2DM	rs13163610	A	*KIAA0825*	**3.22E-10**	NR	NR
Our study	African/T2DM/AADM		C		0.84	1.01	[0.90,1.13]
Our study	African American/T2DM/ARICAA		C		0.26	0.85	[0.64,1.13]
Huang et all.(2011)	Asian/T2DM	rs2038823	C	*HS6ST3*	**4.68E-11**	NR	NR
Our study	African/T2DM/AADM		T		0.84	0.98	[0.78,1.22]
Our study	African American/T2DM/ARICAA		T		0.23	0.78	[0.52,1.17]
Meng et all.(2018)	European/T2DM/GoDARTS	rs3913535	C	*NOX4*	**4.05E-09**	1.55	[1.34,1.79]
Our study	African/T2DM/AADM		C		0.18	0.95	[0.89,1.02]
Our study	African American/T2DM/ARICAA		C		0.68	0.97	[0.82,1.14]

[1] Nominally significant p-values (p<0.05) are shown in bold. [2] ORs are calculated with respect to the minor allele. Abbreviations: chromosome (Chr), Epidemiology of Diabetes Intervention and Control Trail cohort (EDIC), Genetics of kidney in Diabetes cohort (GoKinD), The Wisconsin Epidemiologic Study of Diabetic Retinopathy cohort (WESDR), Genetics of Diabetes Audit and Research in Tayside Scotland (GoDARTS).

Figure S1 Quantile-quantile of p values for PDR.

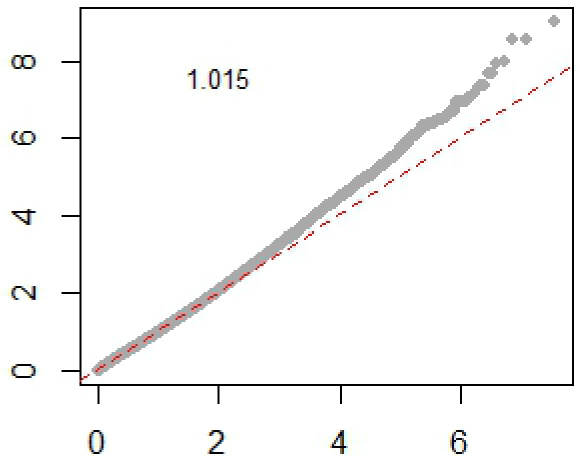

The X-axis represents expected p values and the Y-axis presents observed p values. All p values presented in –log10(p-values in discovery AADM study.

Figure S2. Quantile-quantile plot of p values for PDR stratified by allele frequency

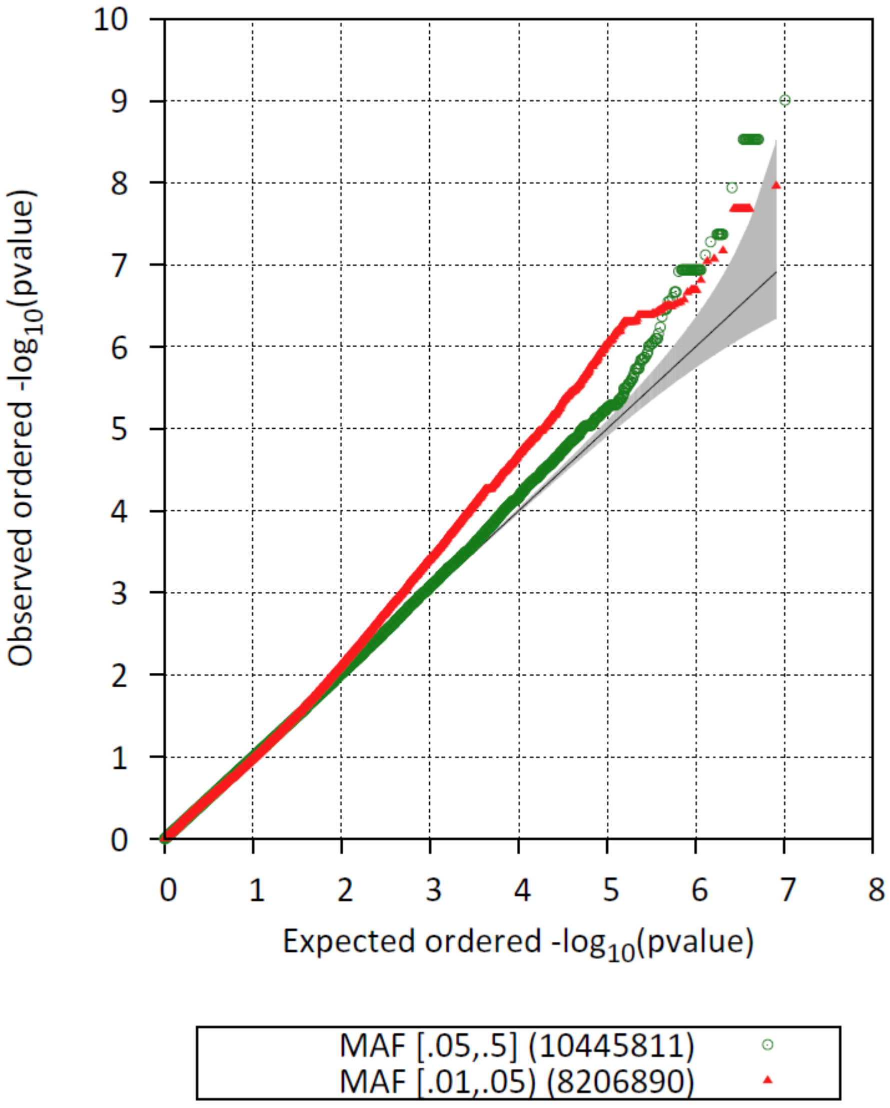

The X-axis represents expected p values and the Y-axis represents observed p values. All p values presented in $-\log_{10}$(p-values) in the AADM study.

Figure S3. Population Structure in AADM and ARIC-AA

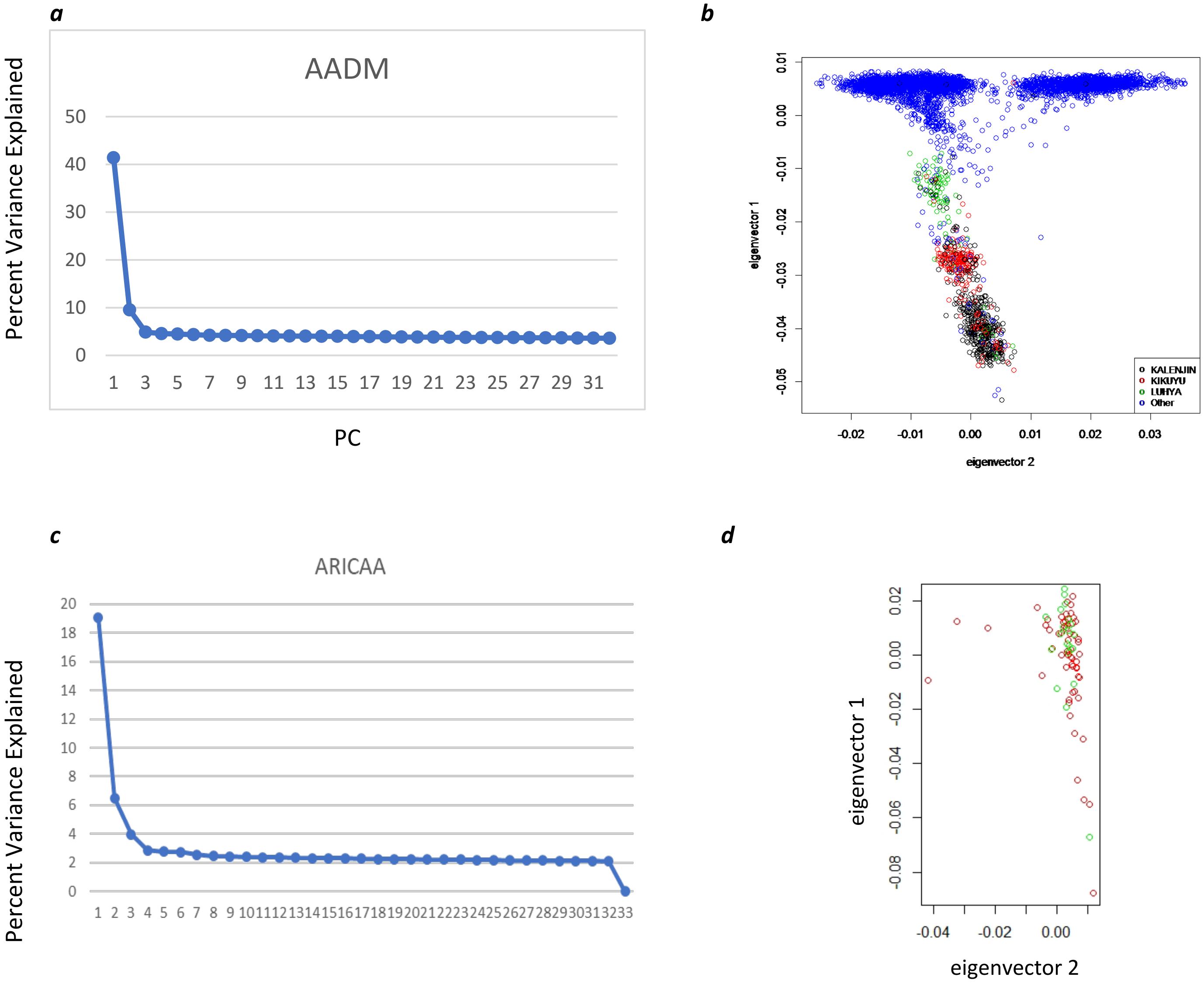

a) Variance explained by PCs in AADM; b) The population structure of AADM; c) Variance explained by PCs in ARIC-AA; d) The population structure of ARIC-AA.
